# Reproducible MS/MS library cleaning pipeline in matchms

**DOI:** 10.1186/s13321-024-00878-1

**Published:** 2024-07-29

**Authors:** Niek F. de Jonge, Helge Hecht, Michael Strobel, Mingxun Wang, Justin J. J. van der Hooft, Florian Huber

**Affiliations:** 1 Bioinformatics Group, Wageningen University & Research, 6708 PB Wageningen, the Netherlands; 2 Faculty of Science, RECETOX, Masaryk University, Kotlářská 2, Brno, Czech Republic; 3 Department of Computer Science and Engineering, University of California Riverside, 900 University Ave., Riverside, CA 92521 USA; 4 Department of Biochemistry, University of Johannesburg, Auckland Park, Johannesburg, 2006 South Africa; 5 Centre for Digitalisation and Digitality, Düsseldorf University of Applied Sciences, 40476 Düsseldorf, Germany

**Keywords:** Library cleaning, Mass spectrometry, Metabolomics, Metadata, Python Package

## Abstract

Mass spectral libraries have proven to be essential for mass spectrum annotation, both for library matching and training new machine learning algorithms. A key step in training machine learning models is the availability of high-quality training data. Public libraries of mass spectrometry data that are open to user submission often suffer from limited metadata curation and harmonization. The resulting variability in data quality makes training of machine learning models challenging. Here we present a library cleaning pipeline designed for cleaning tandem mass spectrometry library data. The pipeline is designed with ease of use, flexibility, and reproducibility as leading principles.

**Scientific contribution**

This pipeline will result in cleaner public mass spectral libraries that will improve library searching and the quality of machine-learning training datasets in mass spectrometry. This pipeline builds on previous work by adding new functionality for curating and correcting annotated libraries, by validating structure annotations. Due to the high quality of our software, the reproducibility, and improved logging, we think our new pipeline has the potential to become the standard in the field for cleaning tandem mass spectrometry libraries.

**Graphical Abstract:**

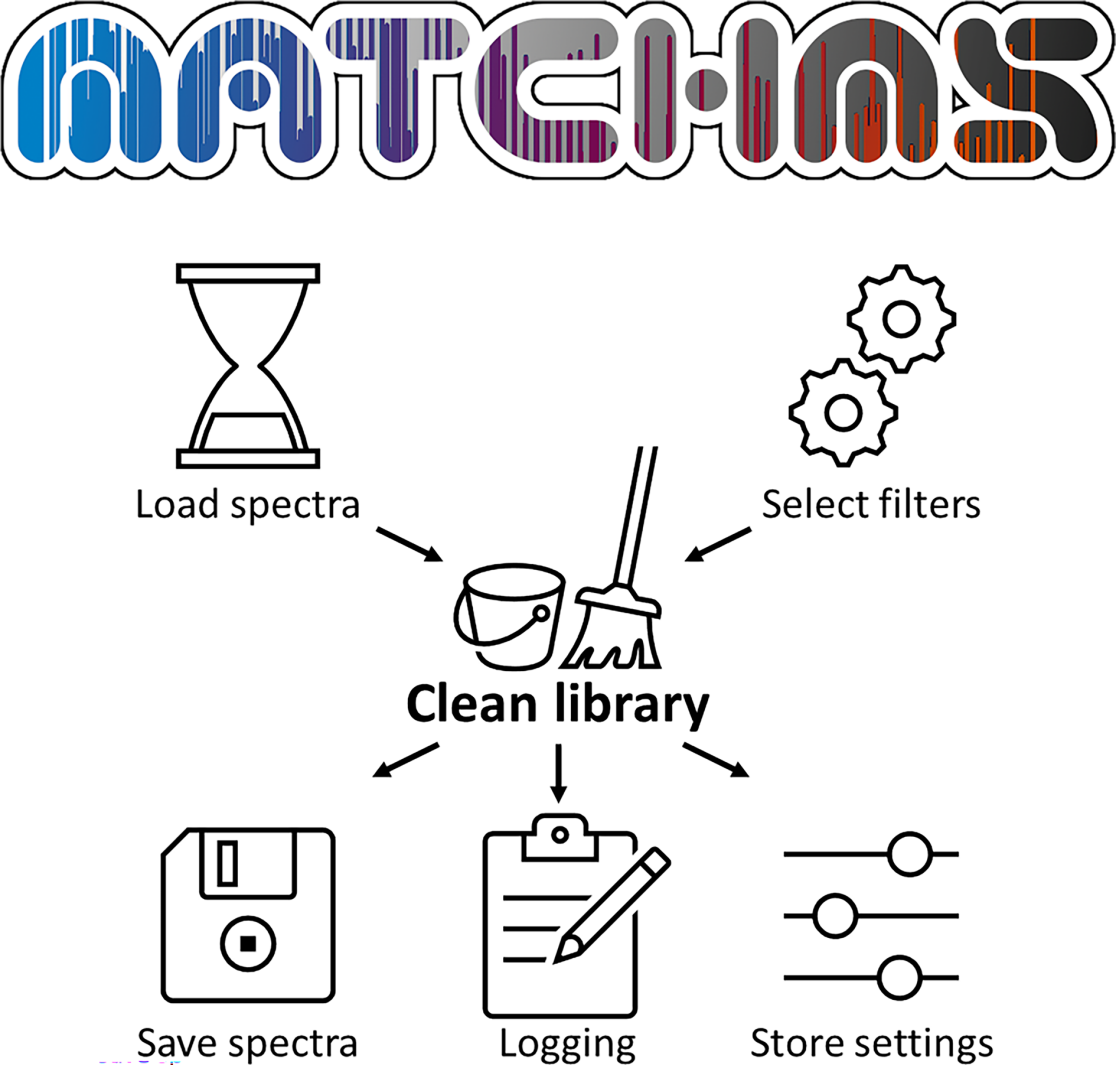

**Supplementary Information:**

The online version contains supplementary material available at 10.1186/s13321-024-00878-1.

## Introduction

Mass spectrometry fragmentation spectra, often referred to as MS/MS spectra or tandem MS spectra, play a pivotal role in molecular structure identification across many fields [14]. However, it remains challenging to link such mass spectra to their corresponding molecular structure [2, 9]. A common approach is to use library matching to identify probable candidates in existing, annotated mass spectral libraries [1, 3]. In addition, several machine-learning approaches have been developed to improve mass spectra annotation [8, 11, 12, 16]. Both for conventional library matching and the training of machine learning models, it is crucial that the annotated library data is correct [9].

Two main types of mass spectral databases are available, public open datasets [15], like the public mass spectral libraries within the GNPS platform [24], and private libraries, like the NIST library (NIST) [20]. Whilst private libraries can be used for library searching if available within a research group, they typically do not share the library spectra in a format that can directly be used to train machine learning models. Even in cases where such libraries are used for model training, the lack of publicly shareable training data makes it hard for others to recreate the models or to analyse potential biases in the used training library. Open-source machine learning models are therefore created by using public libraries.

Whilst we appreciate all the community efforts to build and expand public metabolomics libraries; unfortunately, these public libraries are typically not of high enough, consistent quality to directly use for training machine learning models. There is a lack of standardization of metadata that results in many entries not being computer interpretable without extensive additional metadata harmonization. This hampers an efficient and reliable training of machine learning models and demands considerable efforts related to data preparation. Currently, most machine learning endeavors lack reproducibility and build their cleaning pipelines from the ground up, often without providing sufficiently reusable code. Given the critical nature of library data curation, there is a pressing need for robust and reproducible procedures. Furthermore, a higher-quality mass spectral library will also provide higher-quality hits for library matching and analogue searching.

**Fig. 1:**
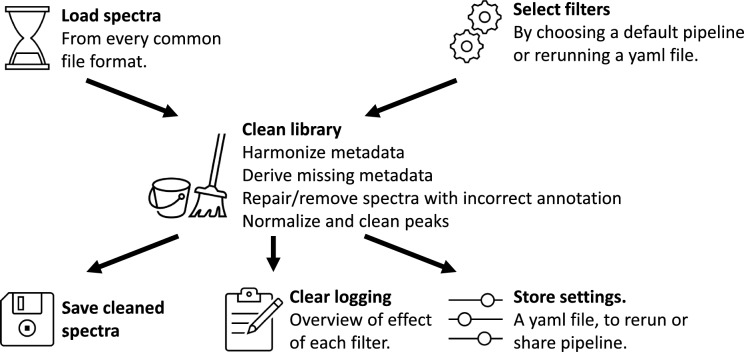
Graphical overview of the library cleaning pipeline

We note that the filtering and cleaning of unannotated mass spectral data, i.e., experimental data, has been supported through modules in tools like MZMine, MSDial, OpenMS for peak picking and matchms for metadata cleaning [17, 21–23]. However, these tools are not yet optimized for the cleaning of library spectra that contain annotations in their metadata. Addressing this gap, we here introduce a comprehensive pipeline for library cleaning within the matchms framework. It encompasses metadata cleaning, peak filtering, intensity normalization, and structure annotation validation through adduct, precursor m/z, and annotation comparison and harmonization. The pipeline is designed to be very easy to run with default settings suitable for common use cases, while offering highly flexible customization options for specific requirements. In addition, it generates reports with a clear overview of the effect each cleaning step had on the library. The final pipeline can easily be shared as a YAML file, ensuring transparency and ease of replication. Figure 1 provides a graphical overview of the pipeline. This pipeline will result in cleaner public libraries, which will improve library searching and will make it easier to train new machine-learning algorithms in mass spectrometry. Here, after detailing the various steps and filters, we demonstrate its use on the GNPS public mass spectral library and share a cleaned version of this library.

## Implementation

The library cleaning pipeline is implemented in the increasingly widely used open-source Python package matchms. The matchms package provides functionality to import, process, clean, and compare mass spectrometry data. The filtering in matchms is structured around individual functions executing a single filter on a single spectrum at a time. In the current work, these filters have been extended to harmonize metadata fields, derive missing data from other metadata and repair or remove incorrect annotations.

Here, we also implemented an easy-to-use automated pipeline including multiple default settings for various use cases, like:

- Basic filters. Runs basic metadata harmonization.
- Default filters. Runs basic metadata harmonization, but also derives missing metadata from other fields, requiring metadata about ionmode and precursor mz and normalizing intensities.
- Library cleaning. Runs all default filters, but in addition repairs errors in the annotations and requires complete annotations after all repairs were run.

These default pipelines are designed to cover most standard use cases. For instance the default filters pipeline is suitable for preprocessing experimental data and the library cleaning pipeline is suitable for preprocessing of library spectra before library matching or machine learning training. However, we are aware that specific use cases might need additional filtering or different parameterization. Therefore, it is possible to create your own pipeline using existing matchms filters, or to add custom-made filters. Since a filter is defined as a function receiving a spectrum and returning a spectrum, users can add arbitrary functions as custom filters as long as they fulfil this condition. To make it easy to create these new pipelines, the pipeline automatically arranges the filters in a predefined global order. This is critical since many filters depend on other processing steps to be effective. For instance, a filter using information from the adduct field should be run after a filter function retrieving the adduct from the compound name field.

### Processing report

A log file is automatically created when running a pipeline on matchms. This log file gives an overview of all the settings and filters used, followed by logging of changes to individual spectra. The logging can be set to different logging levels. To give a clear overview of what changes were made to a mass spectral library, a processing report is generated. This report gives an overview of the number of spectra that were changed in the library by each filter. In this report we differentiate between completely removing spectra, changing the metadata, and changing the peak information. Examples of automatically generated processing reports for library cleaning pipelines can be found in Tables S1 and S2.

### Reproducibility

When running a matchms pipeline created using the “create_workflow” function, it automatically creates a YAML file containing all settings and filters used. This file can be used directly to rerun a cleaning pipeline, making sharing of used workflows very easy and reproducible. Examples of these YAML files can be found on Zenodo [5].

### New filters

New filters were added to those already present within the matchms package to filter out spectra with incorrect annotations or mismatching metadata. In addition, filter functions were created that can repair annotation mistakes, or complete missing metadata from other fields. This ultimately reduces the number of spectra that have to be removed, which results in larger libraries with more diversity. A complete overview of all filters available in matchms can be found at https://matchms.readthedocs.io/en/latest/api/matchms.filtering.html [6].

In the context of our study and the filter description section below, the parent mass refers to the mass of the non-ionized state of a molecule, which is here defined by the monoisotopic mass using the most common isotopes. The precursor m/z is the mass-to-charge-ratio of the ionized molecule selected for fragmentation.

Here below, a list of new filters and their function is provided:

### Derive annotation from compound name

Many annotations in the GNPS library are only compound names without SMILES. This filter derives the canonical SMILES, InChI and InChIKey from PubChem. It will only add SMILES to the annotation if the mass found on PubChem matches with the parent mass in the metadata. Previously found compound names are stored in a file and reused. This significantly reduces the number of times a compound name has to be looked up. For cleaning new libraries these files with common compound names can also be reused.

#### Repair SMILES of salts

Often salts are measured in a mass spectrometer, however, in the mass spectrometer these salts often fall apart into different salts. Resulting in actually measuring only one or a few of the ions added. This results in a mismatch between parent mass and the SMILES match. We repair this, by checking if the parent mass matches any of the separate or combinations of salt ions given in the SMILES. For instance C1=NC2=NC=NC(=C2N1)N.Cl is converted to 1=NC2=NC=NC(=C2N1)N if this matches the parent mass.

#### Repair parent mass is molar mass

A common mistake is that the parent mass is calculated from the molar mass instead of the monoisotopic mass. This filter corrects this.

#### Repair adduct and parent mass based on SMILES

Corrects the adduct of a spectrum based on the SMILES and precursor m/z. Often the adduct is not changed by users from the default [M + H] + to the actual adduct. This results in frequent mismatches between the parent mass and the expected mass for the given SMILES. This filter tries common adducts and selects an adduct that would explain the combination of SMILES and precursor m/z.

#### Repair not matching annotation

If the different metadata fields for an annotation do not match, this filter will try to correct it. Sometimes the SMILES, InChI, or InChIKey do not describe the same compound. Matchms will in these cases keep the metadata field of an annotation that matches with the parent mass and remove the non-matching annotation.

#### Require valid annotation

This filter checks if all the metadata describing an annotation is correct and matches to each other. SMILES, InChI and InChIKey are loaded by RDKit [18] and compared to each other. If one of these is not complete or does not match the spectrum will be removed.

#### Require parent mass match SMILES

This filter removes any spectrum where the monoisotopic mass of the SMILES does not match the parent mass stored in the metadata. This filter is applied after the above-mentioned repair functions. Therefore, only spectra that cannot be repaired by any of these functions will be removed from the library.

#### Require minimum number of high peaks

Many MS2 spectra in the library contain spectra that have almost not been fragmented. This can be an issue, since there might not be enough information in these spectra to do accurate library matching. This filter removes spectra that do not have a minimum number of fragments with an intensity above a set relative intensity.

#### Require matching adduct, precursor m/z and parent mass

The adduct can be used to calculate the parent mass from the precursor m/z. A spectrum is removed, if there is a mismatch between the calculated parent mass and the given parent mass.

#### Require matching adduct and ionmode

Removes spectra where the adduct corresponds to a different ion mode than the one given in the ion mode field.

### Validation

As an example the newly developed pipeline was run on a current version of the GNPS library. The public GNPS library was downloaded on 21-08-2023 (GNPS [13]). We note that we selected the no propagated version to only include experimentally derived mass spectra and annotations. We did run two pipelines with different settings on the GNPS library. For the first pipeline the default settings in the library cleaning pipeline were used and for the second run we used the library cleaning pipeline, without any of the new repair annotation function, to illustrate the effect of these new filters. The cleaned library, the scripts and YAML file with the filters and settings can be found on Zenodo [5]. Matchms version 0.26.4 was used to run these pipelines.

The matchms pipeline was also run on the Massbank library [15], the MoNA library (MoNA. Massbank of North America) [19] and a large MS/MS dataset created by Corinna Brungs et al. [4]. This illustrates the pipeline’s compatibility with different metadata styles and file types. Processing reports for these runs can be found in supplementary Table 3–5.

### Integration into GNPS ecosystem

The matchms pipeline is integrated into the GNPS ecosystem. MS/MS spectral libraries at GNPS are cleaned every 24 h with matchms and made available for download at https://external.gnps2.org/gnpslibrary. Reference MS/MS spectra are additionally enriched by mining the provenance raw data from which each MS/MS library was originally extracted. During this step detailed instrumentation and collision energy metadata were extracted.

### Code quality

Matchms is easily installable through pip or Conda and uses Git for systematic version control, allowing for seamless updates and maintenance. We prioritize robust code quality and stability by maintaining a high unit test coverage. To achieve this, we use SonarCloud’s quality gate, which mandates over 80% code coverage. By using continuous integration, we ensure that matchms works well on Windows, Ubuntu, and MacOS systems, and is compatible with multiple Python versions. By following these good practices, we strive for good code quality and stability of the tool.

## Results

**Fig. 2:**
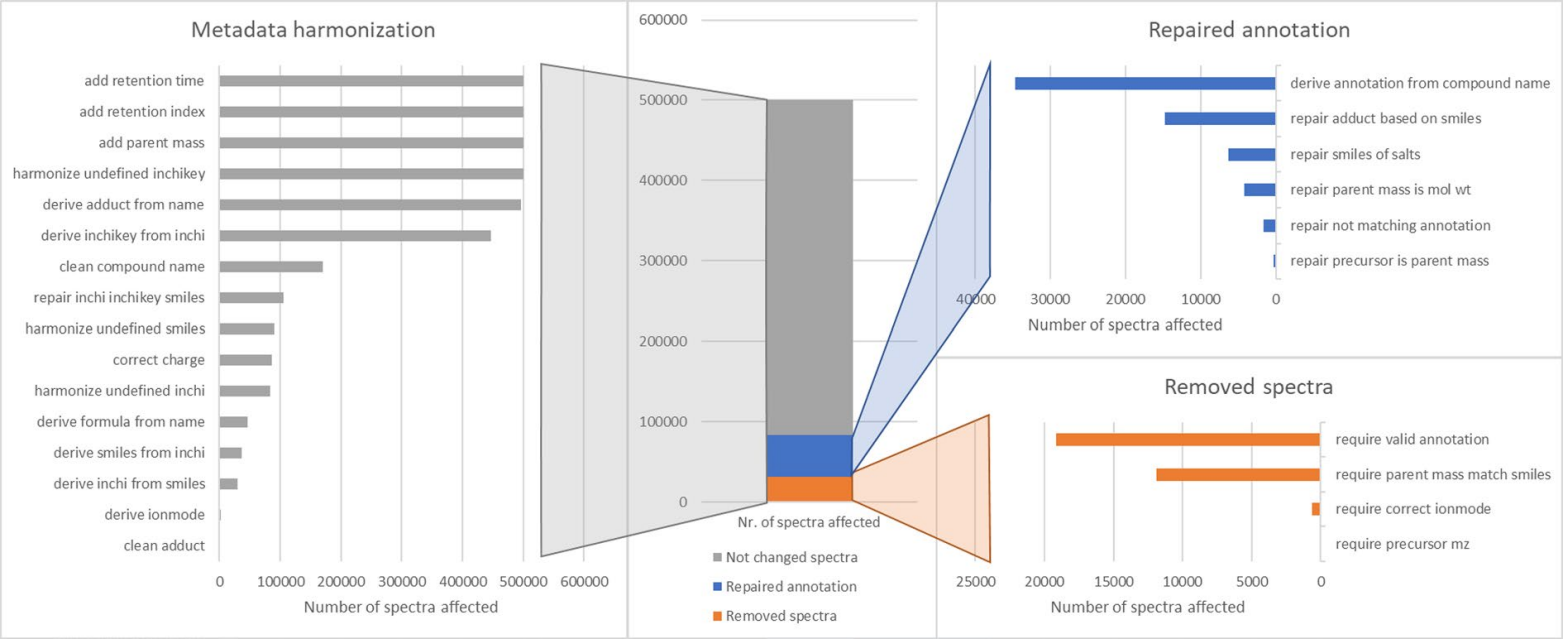
Visualization of the number of spectra in the GNPS library affected by the different filters. The central stacked bar graph splits the spectra into 3 groups. The orange group represents spectra that were completely removed by a filter, since they did not pass a metadata requirement. The blue group represents spectra that were repaired by at least one of the newly added repair functions focused on repairing the annotation. The grey group represents all other spectra, for which the metadata was harmonized, but the annotation or metadata was not affected by the newly added filters

**Fig. 3:**
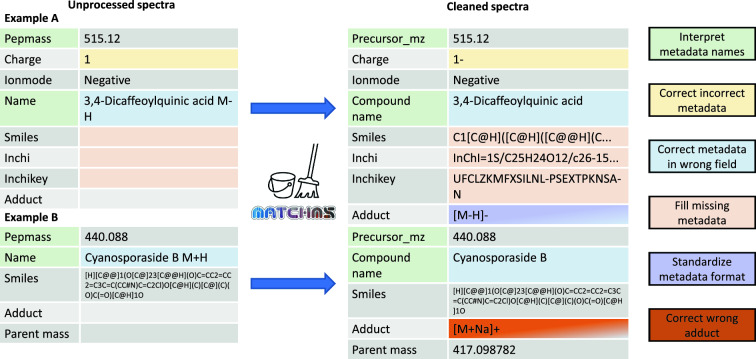
Two real examples of GNPS library spectra that were cleaned in multiple ways by the library cleaning pipeline. The colors indicate the type of changes that were made to the metadata

The new library cleaning pipeline was run on the GNPS public mass spectral library. A YAML file and processing report were automatically generated. The processing report can be found in Supplementary Table 1. Before cleaning, the GNPS library contained 500,569 spectra. Figure 2 gives a visual overview of the number of spectra affected by the different filters. Figure 3 shows two examples of metadata of real spectra stored in the GNPS library and illustrates the effects and importance of metadata curation via matchms.

The library cleaning pipeline was also run without the newly added repair functions focused on repairing annotation. When running this pipeline, without these repair functions, a total of 83,843 spectra were removed, since they did not have a valid annotation or there was a mismatch between parent mass and annotation. In the library cleaning pipeline only 31,758 spectra were removed showing that combined the newly introduced repair functions repaired the metadata of 52,084 spectra. The final cleaned GNPS public mass spectral library contains 448.485 curated mass spectra and is available on Zenodo [5].

The three horizontal bar graphs show how many spectra were affected by each filter within the 3 different groups, i.e., for metadata harmonization (left), repaired annotation (right top), and removed spectra (right bottom). It is important to note that a spectrum can be affected by multiple different filters during a full pipeline run.

Repairing missing metadata carries the risk of incorrectly repairing mass spectra. The error rate was estimated for the filters “derive annotation from compound name” and “repair adduct and parent mass based on SMILES”. GNPS spectra with a valid annotation, a compound name and an adduct matching the ion mode were selected, resulting in a subset containing 413,314 spectra.

After removing the SMILES annotation from the selected spectra the SMILES were derived from the compound name. For 27,6% of the spectra, the SMILES could not be derived from the compound name. Of the spectra that were annotated (72,4%), 1,62% were annotated with a different 2D structure than the original annotation.

The “Repair adduct and parent mass based on SMILES” filter did not derive an adduct for 0,02%. Of the 99,98% of the spectra, 0,024% of the spectra had an incorrect adduct after the filter.

### Running time

Running the machms pipeline with the filters given in Supplementary Table S1 on the GNPS library of 500,569 spectra took 6 h and 45 min.

## Discussion

Current publicly available libraries often have incorrect or incomplete metadata. Here, we present a library cleaning pipeline that can automatically validate annotations and repair mistakes in a reproducible way. The pipeline combines various filter functions to harmonize, derive and repair metadataor discard spectra with incorrect annotations. Rather than simply discarding all mass spectra with mismatching metadata, our pipeline automatically corrects common errors. To reduce the risk of repairing metadata of spectra with incorrect annotations, the pipeline only repairs changes if they are supported by multiple metadata fields. For instance, the SMILES is only derived from PubChem based on a compound name if it matches the given parent mass.

An important aspect of this pipeline is the generation of a detailed processing report with an overview of the effect of each filtering step. This provides insights into metadata deficiencies in a library and the pipeline’s effectiveness. Examples of such processing reports can be found in Table S1 and Table S2. Another important goal was to increase the reproducibility of library cleaning, harmonization, and processing. Hence, our pipeline automatically creates a YAML file. This YAML file gives a clear human-readable and computer-readable record of all the performed filter steps and can be used as direct input to rerun the pipeline. This facilitates sharing and replication of such mass spectral filtering processes.

The library cleaning pipeline is implemented in matchms. By extending the package, we thereby reuse existing functionality (e.g., loading mass spectra of diverse formats and standardizing metadata fields) saving implementation time and fostering collaboration. Matchms has an increasing active user base, a team of multiple developers, and a high standard for code quality. We expect that the here described developments will further stimulate its use throughout the metabolomics community and the mass spectrometry community in general.

This pipeline repairs errors in the metadata for spectra that are already uploaded to public libraries. A more desirable approach to improve mass spectral library quality in the future would be to curate spectra during the uploading of new library spectra, instead of repairing mistakes after the fact. One of the main challenges here is that this is mostly relying on voluntary work; hence, such a process should not be too time-consuming. Therefore, there is a need for easy and fast methods for automatic curation while uploading to public mass spectral libraries, ultimately improving their metadata quality and coverage. We expect that the here described developments can contribute to such a validator tool, that will also automatically fill metadata fields that can be propagated from other fields, and flag internal metadata inconsistencies.

While the current filters address many annotation inaccuracies, they currently still lack plausibility checks that consider both metadata and measured fragments. Wrong chemical annotations that are consistent with the measured mass, for instance, will go unnoticed in the current pipeline. Future expansions might include filters that check if the fragments match the given annotation. Additionally, we note that future use cases may rely on other metadata fields that are not yet cleaned by the current matchms filters. For instance, the instrument type or collision energies could be important fields that might require additional standardization. The modular and flexible structure of matchms makes it straightforward to add such filters to the matchms pipeline in the future.

## Conclusions

Overall, our mass spectral library cleaning pipeline aims to simplify library cleaning and making the process reproducible, resulting in higher-quality spectral libraries. The ease of use combined with the flexibility also allows for future development that can further improve and standardize current practices in mass spectrometry-based metabolomics. Our pipeline is expected to improve the quality of public mass spectral libraries and thereby improve the annotation results of library matching and analogue searching, as well as the future performance of machine learning models.

### Supplementary Information

Supplementary file 1.

## Data Availability

All code is available on GitHub [7] and Zenodo [10]. The settings used for the GNPS public mass spectral libraries use case, the logging, and the cleaned libraries can be found on Zenodo [5]. A tutorial for using matchms can be found on https://matchms.github.io/matchms-docs/intro.html. The full matchms source code is provided on the project home page: https://github.com/matchms/matchms. Matchms is automatically tested to run on MacOS, Ubuntu and Windows systems. In its current version, matchms runs on Python 3.9, 3.10, 3.11 or 3.12. Other dependencies can be found in https://github.com/matchms/matchms/blob/master/pyproject.toml. Matchms is licensed under the Apache License 2.0
