# Supplementary material for: Reproducible MS/MS library cleaning pipeline in matchms
Source: J Cheminform. 2024 Jul 29;16:88. doi: 10.1186/s13321-024-00878-1 (PMC11285329; doi:10.1186/s13321-024-00878-1)
Supplement: Supplementary file 1 [file 13321_2024_878_MOESM1_ESM.docx]

Supplementary information

| **filter** | **removed spectra** | **changed metadata** | **changed mass spectrum** |
| --- | --- | --- | --- |
| add_parent_mass | 0 | 500519 | 0 |
| add_retention_index | 0 | 500569 | 0 |
| add_retention_time | 0 | 500569 | 0 |
| clean_adduct | 0 | 633 | 0 |
| clean_compound_name | 0 | 170095 | 0 |
| correct_charge | 0 | 85776 | 0 |
| derive_adduct_from_name | 0 | 496399 | 0 |
| derive_annotation_from_compound_name | 0 | 34571 | 0 |
| derive_formula_from_name | 0 | 46915 | 0 |
| derive_inchi_from_smiles | 0 | 30143 | 0 |
| derive_inchikey_from_inchi | 0 | 446730 | 0 |
| derive_ionmode | 0 | 1041 | 0 |
| derive_smiles_from_inchi | 0 | 37013 | 0 |
| harmonize_undefined_inchi | 0 | 83009 | 0 |
| harmonize_undefined_inchikey | 0 | 500519 | 0 |
| harmonize_undefined_smiles | 0 | 90214 | 0 |
| normalize_intensities | 0 | 0 | 458606 |
| repair_adduct_and_parent_mass_based_on_smiles | 0 | 8448 | 0 |
| repair_inchi_inchikey_smiles | 0 | 106092 | 0 |
| repair_not_matching_annotation | 0 | 1665 | 0 |
| repair_parent_mass_is_molar_mass | 0 | 1186 | 0 |
| repair_smiles_of_salts | 0 | 6325 | 0 |
| require_correct_ionmode | 622 | 0 | 0 |
| require_matching_adduct_and_ionmode | 393 | 0 | 0 |
| require_matching_adduct_precursor_mz_parent_mass | 1060 | 0 | 0 |
| require_parent_mass_match_smiles | 19787 | 0 | 0 |
| require_precursor_mz | 50 | 0 | 0 |
| require_valid_annotation | 19231 | 0 | 0 |
| require_correct_ms_level | 0 | 0 | 0 |
| make_charge_int | 0 | 0 | 0 |
| add_compound_name | 0 | 0 | 0 |
| interpret_pepmass | 0 | 0 | 0 |
| add_precursor_mz | 0 | 0 | 0 |

**Table S1:** Processing Report of the library cleaning pipeline. The GNPS library (GNPS Library 2023) consisting of 500569 was cleaned by the pipeline. For each filter the number of spectra that were affected by the filter is given. In total the pipeline removed 41143 spectra.

| **filter** | **removed spectra** | **changed metadata** | **changed mass spectrum** |
| --- | --- | --- | --- |
| require_parent_mass_match_smiles | 29242 | 0 | 0 |
| require_valid_annotation | 53892 | 0 | 0 |
| require_precursor_mz | 50 | 0 | 0 |
| require_correct_ionmode | 659 | 0 | 0 |
| derive_adduct_from_name | 0 | 496399 | 0 |
| add_retention_index | 0 | 500569 | 0 |
| add_retention_time | 0 | 500569 | 0 |
| correct_charge | 0 | 86398 | 0 |
| harmonize_undefined_inchikey | 0 | 500519 | 0 |
| harmonize_undefined_inchi | 0 | 83009 | 0 |
| add_parent_mass | 0 | 500519 | 0 |
| derive_inchi_from_smiles | 0 | 30141 | 0 |
| derive_inchikey_from_inchi | 0 | 446730 | 0 |
| harmonize_undefined_smiles | 0 | 90214 | 0 |
| clean_adduct | 0 | 633 | 0 |
| repair_inchi_inchikey_smiles | 0 | 106092 | 0 |
| derive_smiles_from_inchi | 0 | 37012 | 0 |
| derive_formula_from_name | 0 | 46915 | 0 |
| clean_compound_name | 0 | 170095 | 0 |
| derive_ionmode | 0 | 1663 | 0 |
| normalize_intensities | 0 | 0 | 415975 |
| make_charge_int | 0 | 0 | 0 |
| add_compound_name | 0 | 0 | 0 |
| interpret_pepmass | 0 | 0 | 0 |
| add_precursor_mz | 0 | 0 | 0 |

**Table S2:** Processing Report of the library cleaning pipeline, without running any of the new repair functions. The GNPS library (GNPS Library 2023) consisting of 500569 was cleaned by the pipeline. For each filter the number of spectra that were affected by the filter is given. In total the pipeline removed 83843 spectra.

| **filter** | **Removed spectra** | **Changed metadata** | **Changed mass spectrum** |
| --- | --- | --- | --- |
| add_parent_mass | 0 | 177390 | 0 |
| add_retention_index | 0 | 177390 | 0 |
| clean_adduct | 0 | 4075 | 0 |
| clean_compound_name | 0 | 3642 | 0 |
| correct_charge | 0 | 83839 | 0 |
| derive_adduct_from_name | 0 | 6 | 0 |
| derive_formula_from_name | 0 | 349 | 0 |
| derive_inchikey_from_inchi | 0 | 177385 | 0 |
| harmonize_undefined_inchikey | 0 | 177390 | 0 |
| normalize_intensities | 0 | 0 | 177332 |
| repair_adduct_and_parent_mass_based_on_smiles | 0 | 20448 | 0 |
| repair_parent_mass_is_molar_mass | 0 | 10 | 0 |
| require_parent_mass_match_smiles | 53 | 0 | 0 |
| require_valid_annotation | 5 | 0 | 0 |
| require_correct_ms_level | 0 | 0 | 0 |
| make_charge_int | 0 | 0 | 0 |
| add_compound_name | 0 | 0 | 0 |
| interpret_pepmass | 0 | 0 | 0 |
| add_precursor_mz | 0 | 0 | 0 |
| add_retention_time | 0 | 0 | 0 |
| derive_ionmode | 0 | 0 | 0 |
| require_precursor_mz | 0 | 0 | 0 |
| harmonize_undefined_inchi | 0 | 0 | 0 |
| harmonize_undefined_smiles | 0 | 0 | 0 |
| repair_inchi_inchikey_smiles | 0 | 0 | 0 |
| derive_annotation_from_compound_name | 0 | 0 | 0 |
| derive_smiles_from_inchi | 0 | 0 | 0 |
| derive_inchi_from_smiles | 0 | 0 | 0 |
| repair_smiles_of_salts | 0 | 0 | 0 |
| repair_not_matching_annotation | 0 | 0 | 0 |
| require_correct_ionmode | 0 | 0 | 0 |
| require_matching_adduct_precursor_mz_parent_mass | 0 | 0 | 0 |
| require_matching_adduct_and_ionmode | 0 | 0 | 0 |

**Table S3:** Processing Report of the library cleaning pipeline, run on the library created by Corinna Brungs et al. (Brungs et al. 2024). The library consisting of 177390 was cleaned by the pipeline. For each filter the number of spectra that were affected by the filter is given. In total the pipeline removed 58 spectra.

| **filter** | **Removed spectra** | **Changed metadata** | **Changed mass spectrum** |
| --- | --- | --- | --- |
| add_parent_mass | 0 | 140306 | 0 |
| add_retention_index | 0 | 143597 | 0 |
| add_retention_time | 0 | 76263 | 0 |
| clean_adduct | 0 | 5000 | 0 |
| clean_compound_name | 0 | 10153 | 0 |
| correct_charge | 0 | 143597 | 0 |
| derive_adduct_from_name | 0 | 176 | 0 |
| derive_annotation_from_compound_name | 0 | 19507 | 0 |
| derive_formula_from_name | 0 | 41 | 0 |
| derive_inchi_from_smiles | 0 | 12624 | 0 |
| derive_inchikey_from_inchi | 0 | 5 | 0 |
| derive_ionmode | 0 | 137813 | 0 |
| derive_smiles_from_inchi | 0 | 38276 | 0 |
| harmonize_undefined_inchi | 0 | 34692 | 0 |
| harmonize_undefined_inchikey | 0 | 53 | 0 |
| harmonize_undefined_smiles | 0 | 60289 | 0 |
| normalize_intensities | 0 | 0 | 129046 |
| repair_adduct_and_parent_mass_based_on_smiles | 0 | 1520 | 0 |
| repair_inchi_inchikey_smiles | 0 | 77 | 0 |
| repair_not_matching_annotation | 0 | 43 | 0 |
| repair_smiles_of_salts | 0 | 855 | 0 |
| require_correct_ionmode | 2531 | 0 | 0 |
| require_correct_ms_level | 17396 | 0 | 0 |
| require_matching_adduct_precursor_mz_parent_mass | 5020 | 0 | 0 |
| require_parent_mass_match_smiles | 316 | 0 | 0 |
| require_precursor_mz | 3291 | 0 | 0 |
| require_valid_annotation | 2616 | 0 | 0 |
| make_charge_int | 0 | 0 | 0 |
| add_compound_name | 0 | 0 | 0 |
| interpret_pepmass | 0 | 0 | 0 |
| add_precursor_mz | 0 | 0 | 0 |
| require_matching_adduct_and_ionmode | 0 | 0 | 0 |
| repair_parent_mass_is_molar_mass | 0 | 0 | 0 |

**Table S4:** Processing Report of the library cleaning pipeline, run on the MoNA Library (MoNA. Massbank of north america. ). The library consisting of 160993 was cleaned by the pipeline. For each filter the number of spectra that were affected by the filter is given. In total the pipeline removed 31170 spectra.

| **filter** | **Removed spectra** | **Changed metadata** | **Changed mass spectrum** |
| --- | --- | --- | --- |
| add_parent_mass | 0 | 92402 | 0 |
| add_retention_index | 0 | 96350 | 0 |
| add_retention_time | 0 | 96350 | 0 |
| clean_adduct | 0 | 241 | 0 |
| clean_compound_name | 0 | 58 | 0 |
| correct_charge | 0 | 96350 | 0 |
| derive_formula_from_name | 0 | 53 | 0 |
| derive_inchikey_from_inchi | 0 | 204 | 0 |
| derive_smiles_from_inchi | 0 | 16 | 0 |
| harmonize_undefined_inchi | 0 | 437 | 0 |
| harmonize_undefined_inchikey | 0 | 641 | 0 |
| harmonize_undefined_smiles | 0 | 363 | 0 |
| normalize_intensities | 0 | 0 | 86215 |
| repair_adduct_and_parent_mass_based_on_smiles | 0 | 5000 | 0 |
| repair_inchi_inchikey_smiles | 0 | 90 | 0 |
| repair_not_matching_annotation | 0 | 16 | 0 |
| repair_parent_mass_is_molar_mass | 0 | 15093 | 0 |
| repair_smiles_of_salts | 0 | 2 | 0 |
| require_correct_ms_level | 21382 | 0 | 0 |
| require_matching_adduct_precursor_mz_parent_mass | 5512 | 0 | 0 |
| require_parent_mass_match_smiles | 238 | 0 | 0 |
| require_precursor_mz | 3948 | 0 | 0 |
| require_valid_annotation | 437 | 0 | 0 |
| make_charge_int | 0 | 0 | 0 |
| add_compound_name | 0 | 0 | 0 |
| derive_adduct_from_name | 0 | 0 | 0 |
| interpret_pepmass | 0 | 0 | 0 |
| add_precursor_mz | 0 | 0 | 0 |
| derive_ionmode | 0 | 0 | 0 |
| derive_annotation_from_compound_name | 0 | 0 | 0 |
| derive_inchi_from_smiles | 0 | 0 | 0 |
| require_correct_ionmode | 0 | 0 | 0 |
| require_matching_adduct_and_ionmode | 0 | 0 | 0 |

**Table S5:** Processing Report of the library cleaning pipeline, run on the MassBank Library (Horai et al. 2010). The library consisting of 117732 was cleaned by the pipeline. For each filter the number of spectra that were affected by the filter is given. In total the pipeline removed 31517 spectra.

References

Brungs C, Schmid R, Heuckeroth S, Mazumdar A, Drexler M, Šácha P, Dorrestein PC, Petras D, Nothias L-F, Nencka R (2024) Efficient generation of open multi-stage fragmentation mass spectral libraries.

GNPS Library. (2023). <https://gnps-external.ucsd.edu/gnpslibrary/ALL_GNPS_NO_PROPOGATED.mgf> Accessed 21-08-2023

Horai H, Arita M, Kanaya S, Nihei Y, Ikeda T, Suwa K, Ojima Y, Tanaka K, Tanaka S, Aoshima K (2010) MassBank: a public repository for sharing mass spectral data for life sciences. Journal of mass spectrometry 45 (7):703-714

MoNA. Massbank of north america. . <https://mona.fiehnlab.ucdavis.edu/>. Accessed 27-05-2024
